# Daytime Sleepiness in Parkinson’s Disease: A Reappraisal

**DOI:** 10.1371/journal.pone.0107278

**Published:** 2014-09-08

**Authors:** Valérie Cochen De Cock, Sophie Bayard, Isabelle Jaussent, Mahmoud Charif, Magda Grini, Muriel Croisier Langenier, Huan Yu, Regis Lopez, Christian Geny, Bertrand Carlander, Yves Dauvilliers

**Affiliations:** 1 Unité des troubles du sommeil, Hôpital Gui de Chauliac, Montpellier, France; 2 Service de Neurologie, Hôpital Gui de Chauliac, Montpellier, France; 3 Inserm, U1061, Université Montpellier I, Montpellier, France; 4 Department of Neurology, Shanghai Huashan Hospital, Shanghai Fudan University, Shanghai, China; Biogen Idec, United States of America

## Abstract

**Background:**

Excessive daytime sleepiness is a frequent complaint in Parkinson’s disease (PD); however the frequency and risk factors for objective sleepiness remain mostly unknown. We investigated both the frequency and determinants of self-reported and objective daytime sleepiness in patients with Parkinson’s disease (PD) using a wide range of potential predictors.

**Methods:**

One hundred and thirty four consecutive patients with PD, without selection bias for sleep complaint, underwent a semi-structured clinical interview and a one night polysomnography followed by a multiple sleep latency test (MSLT). Demographic characteristics, medical history, PD course and severity, daytime sleepiness, depressive and insomnia symptoms, treatment intake, pain, restless legs syndrome, REM sleep behaviour disorder, and nighttime sleep measures were collected. Self-reported daytime sleepiness was defined by an Epworth Sleepiness Scale (ESS) score above 10. A mean sleep latency on MSLT below 8 minutes defined objective daytime sleepiness.

**Results:**

Of 134 patients with PD, 46.3% had subjective and only 13.4% had objective sleepiness with a weak negative correlation between ESS and MSLT latency. A high body mass index (BMI) was associated with both ESS and MSLT, a pain complaint with ESS, and a higher apnea/hypopnea index with MSLT. However, no associations were found between both objective and subjective sleepiness, and measures of motor disability, disease onset, medication (type and dose), depression, insomnia, restless legs syndrome, REM sleep behaviour disorder and nighttime sleep evaluation.

**Conclusion:**

We found a high frequency of self-reported EDS in PD, a finding which is however not confirmed by the gold standard neurophysiological evaluation. Current treatment options for EDS in PD are very limited; it thus remains to be determined whether decreasing pain and BMI in association with the treatment of sleep apnea syndrome would decrease significantly daytime sleepiness in PD.

## Introduction

Since initial descriptions of sleep attacks at the wheel in patients with Parkinson’s Disease (PD) treated with dopamine agonists, [1] many cross-sectional studies have reported a high frequency (up to 50%) of self-reported excessive daytime sleepiness (EDS) in PD [2]–[11]. In contrast, only a few studies have specifically investigated objective daytime sleepiness in PD using the gold standard multiple sleep latency test (MSLT) with highly variable results [3], [7]–[14]. The large variability between studies as to the prevalence of EDS in PD can be partly attributed to the tools used to define EDS and its cut-off, medication intake, comorbidities, and the targeted PD population [2]–[11]. Although still controversial, the most frequently associated factors with the complaint of EDS in PD are dopaminergic drugs (the dose rather than the specific type of dopamine agonist), [5], [6], [10], [12]–[14] disease severity and duration, [10], [14] reduced activity of daily living, [2], [11], [15] and nighttime sleep problems including the apnea/hypopnea index [10], [12], [14]. However, the frequency and determinants of objective sleepiness in PD remain mostly unknown, due to differences in study populations included either with EDS or unselected for sleep problems, low sample sizes, and various associated potential confounding factors. In addition, the frequency of pathological daytime REM sleep propensity in PD and its predictors remain also questionable, especially in unselected patients for sleep problems or hallucinations [3], [9], [10].

We aimed in this study to assess the frequency of both self-reported EDS using the Epworth Sleep Scale (ESS) and objective EDS using the gold standard MSLT in a large population of unselected consecutive patients with PD. We explored a wide range of potential predictors of either subjective or objective EDS, such as demographic characteristics, medical history, PD course and severity, levodopa equivalent dose and time of intake, other psychotropic drugs, comorbid clinical sleep problems (restless legs syndrome-RLS, REM sleep behaviour disorder (RBD) and insomnia symptoms), cognitive problems, depressive symptoms, pain and nighttime sleep characteristics (sleep time, sleep disordered breathing and periodic leg movements) using polysomnography assessment.

## Methods

### Patients

The study was approved by the research scientific committee (University Hospital, Montpellier, France) and all participants gave written informed consent prior to the study.

From January 2001 to January 2011, 134 consecutive patients with PD unselected for any sleep complaint underwent a semi-structured clinical interview and one night polysomnography followed by a MSLT. Patients met the criteria for definite idiopathic PD [16]. Data concerning demographic characteristics, body mass index (BMI; normal weight: BMI<25 kg/m2; overweight between 25 and 30, and obese ≥30), medical history, PD course, and treatment (with particular attention to levodopa, dopamine agonists, analgesics and psychoactive drugs) were collected during a face-to-face interview. The levodopa equivalent dose taken before 12 AM, between 12 AM and 7 PM, and after 7 PM was detailed. Motor disability using the Unified Parkinson’s Disease Rating Scale motor examination (UPDRS-III) and the Hoehn and Yahr stage were assessed in all patients at the optimal medication (‘on’ condition). All PD patients performed a Mini Mental State Examination. None met the diagnostic criteria of dementia.

Subjective sleepiness was defined by an ESS score above 10 [17]. Restless Legs Syndrome was diagnosed if patients fulfilled the 4 clinical criteria defined by the International Restless Legs Syndrome study group. Clinical REM-sleep behaviour disorder was defined when the bed partner reported significant, purposeful limb or body movements and when these movements were associated with a dream recall upon awakening. Depressive symptoms were explored with the Beck Depression Inventory and moderate or severe depressive symptoms were defined by a score above 19. Insomnia was evaluated using the Insomnia Severity Index and sub-threshold insomnia was defined by a score above 8 [18]. Pain was evaluated using the West Haven-Yale Multidimensional Pain Inventory (WHYMPI) [19]. This inventory examines pain complaint during the previous week and the impact of pain on the patient’s life, the responses of others to the patient’s complaint of pain, and the extent to which the patient participates in common daily activities.

### Polysomnography recordings

All participants had an audio and video PSG during a single night in the sleep unit. Sleep recording included several EEG leads (C3/A2, Fp1/T1, T1/O1, O1/C3, C4/A1, Fp2/T2, T2/O2, O2/C4), right and left electro-oculogram, electromyography of chin and tibialis anterior muscles, nasal pressure monitoring through a cannula, thoracic and abdominal belts for assessing respiratory efforts, electrocardiography and pulse oxymetry. Sleep stages, microarousals, respiratory events, periodic leg movements during sleep (PLMS) were scored by visual inspection according to standard criteria [20]. Severe sleep apnea syndrome (SAS) was defined as an apnea/hypopnea index (AHI) above 30/hour.

On the day following the nocturnal recording standard, MSLT was performed starting two hours after waking up [21]. Five naps of twenty minutes duration were recorded every two hours between 9∶00 to 17∶00. For each recording, the patients were allowed 20 min to fall asleep. If they did fall asleep, they were allowed to sleep an additional 15 min and were then awaked. Sleep onset was determined by the first epoch of any stage of sleep according to standard criteria [20]. A mean sleep latency from the 5 naps below 8 minutes defined objective daytime sleepiness. Sleep onset REM period (SOREMP) was defined as the presence of REM sleep within the 15 minutes following sleep onset at each test.

### Statistical analysis

Categorial variables are presented as percentages, and quantitative variables as medians with ranges or means with and standard deviations (SD), depending on the normality of their distribution. Normality was tested according to Shapiro-Wilk’s test. Spearman’s rank order correlations were applied to determine associations between two continuous variables. Univariate comparisons between patients with PD with and without either self-reported or objective sleepiness were performed using logistic regression analysis. Associations were quantified using odds ratios (OR) and 95% confidence intervals (CI). Sociodemographic variables associated with the two groups (at p<0.10) were included in multivariate logistic regression models to estimate adjusted odd ratios for clinical and sleep variables. When appropriate, the interaction terms were tested using the Wald χ^2^ test given by the logistic regression model. Significance level was set at p<0.05. Statistical analyses were performed using SAS software, version 9.2 (SAS Institute, Cary, NC, USA).

## Results

### Patient characteristics

One hundred and thirty four patients with PD (96 males, mean age 65.9 years old, range 45 to 86) were included. The mean (SD) disease duration at the time of the study was 8.1±5.8 years. Hoehn and Yahr staging 2.2±0.8 and UPDRS-III scale 21.9±12.8. More than one third of the patients (35.8%) were overweight and 11.9% were obese. The percentage of patients on one dopamine agonist was 72.5%, while that of patients using two or three dopamine agonists was 6.9% and 0.8%, respectively. Of the sample, 86% were taking levodopa, with a mean equivalent daily dose of 649±354 mg. Thirty patients (22.4%) were taking antidepressants and 34 (25.6%) benzodiazepines. Overall 62 (46.2%) patients with PD self-reported daytime sleepiness, and 27.4% severe daytime sleepiness (ESS>16). Clinical assessment of RLS was present in 31.3% of patients, clinical RBD in 47.3%, insomnia symptoms (ISI≥8) in 83.9%, a complaint of pain in 76% based on the WHYMPI questionnaire, and depressive symptoms (BDI≥19) in 27.7%.

Polysomnographic characteristics showed low sleep efficiency (64.5±17.0) with 20.9% of patients having a level below 50%. Mean PLMS and PLMS associated with microarousals indexes were respectively 16.6±30.7 and 2.3±5.0, with 27.8% of patients having a PLMS index above 15. Mean AHI was 14.04±1.88 and severe SAS (AHI>30/hour) was reported in only 17.9% of patients.

The overall mean MSLT was 14.4±5.2 with only 13.4% of patients below the cut-off of 8 minutes. Only three patients had one SOREMP on the MSLT and none had two or more SOREMPs.

### Comparisons between PD patients with and without complaint of EDS

Patients with ESS>10 had a higher BMI than patients without EDS (Table 1). Subsequent analyses were thus adjusted for BMI. No significant between-group differences were found for motor disability, PD onset, disease duration, treatment (type, dose and daytime distribution), depressive symptoms, cognitive impairment, or comorbid sleep disorders. In contrast, the presence of pain complaint was more frequent in sleepy patients (OR = 4.16; 95% CI = [1.24–13.9], p = 0.02), without any differences according to analgesic intake (type or dose), pain severity, affective distress, requirement of support or interference with daily living. No significant interaction was found between pain and BMI for the presence of EDS in PD patients (Wald χ^2^ test, p = 0.55). To determine which variables between BMI and pain were independently associated with EDS, the two variables were entered together in the same logistic regression. BMI was not associated with EDS after adjustment for pain.

**Table 1:** Demographic and Clinical characteristics of patients with Parkinson’s disease with subjective sleepiness (Epworth Sleepiness Scale-ESS>10) and without subjective sleepiness (ESS≤10).

	Subjective sleepiness			
Variables	ESS>10 N = 62	ESS≤10 N = 72	Global p	Global p(1)
Age (in years)	65.5±8.3	66.3±8.7	0.58	
Sex *(% male)*	74.2	69.4	0.54	
BMI (kg/m^2^)	26.0±4.4	24.4±3.6	**0.02**	
Motor disability				
UPDRS-III/108	22.2±13.1	21.6±12.8	0.82	0.80
Hoehn and Yahr	2.1±0.8	2.3±0. 9	0.25	0.26
Age at onset (in years)	57.7±9.3	58.0±9.8	0.84	0.77
Disease duration (in years)	7.8±6.0	8.3±5.6	0.64	0.89
Restless legs syndrome (%)	30.6	31.9	0.87	0.88
Clinical RBD (%)	42.4	51.4	0.31	0.20
Insomnia severity index	13.1±5.3	12.9±5.3	0.88	0.95
BDI score ≥19 (%)	32.1	24.2	0.34	0.26
Depression in the past (%)	27.0	26.0	0.91	0.91
MMSE score ≥26 (%)	89.7	87.1	0.66	0.74
Pain complaint (%)	88.6	65.2	**0.02**	**0.02**
Levodopa equivalent dose (mg/day)	762 [100–1850]	743 [67–2158]	0.86	0.90
intake before 12 AM	250 [0–705]	250 [25–750]	0.64	0.54
intake between 12 AM and 7 PM	250 [0–600]	266 [0–983]	0.54	0.57
intake after 7 PM	200 [0–533]	200 [0–550]	0.97	0.95
Dopamine agonists intake (%)	80.0	80.3	0.99	0.72
Antidepressants intake (%)	19.3	25.0	0.44	0.42
Benzodiazepines intake (%)	19. 7	30. 6	0.15	0.29

(1) after adjustment for body mass index; continuous variables are expressed by means ± standard deviation (SD) or median [Min–Max] according to the normality of the distribution.

Comparing patients with and without pain, no significant differences were observed according to disease severity, levodopa equivalent daily dose, use of antidepressants or benzodiazepine, depressive symptoms, total sleep time or sleep efficiency. However, the percentage of patients with ESS>10 was higher in the group of patients with pain (20%) than in the group of patients without pain (4%) (p = 0.02).

Comparison of nighttime sleep between PD patients with and without self-reported EDS revealed no significant differences (Table 2). Patients with ESS>10 had a shorter MSLT latency than patients without (13.7 [2.8–20.0] vs 17.4 [4.4–20.0]; p = 0.007). This, association persists after adjustment for BMI (p = 0.03) (Table 2). If we categorized the mean sleep latency variable into two groups (<8 min vs ≥8 min), the percentage of patients <8 min tended to be higher in the group of patients with ESS≤10 than in the group with ESS>10 (p = 0.07); however this borderline association did not persist after adjustment for BMI (Table 2). Only a slight negative correlation found between ESS and MSLT latency (r = −0.28 p = 0.001). Finally, the percentage of patients with one SOREMP did not differ between patients with and without complaint of sleepiness (3.2 vs 1.4%).

**Table 2:** Night-time sleep and Multiple Sleep Latency Tests measures of patients with Parkinson’s disease with subjective sleepiness (Epworth Sleepiness Scale-ESS>10) compared to patients without subjective sleepiness (ESS≤10).

	Subjective sleepiness			
Variables	ESS>10 N = 62	ESS≤10 N = 72	*p*	*p* (1)
Night-time sleep				
Total sleep time (minutes)	339.5 [69–456]	294 [80–447]	0.05	0.08
Sleep efficiency	69 [17–95]	63 [16–89]	0.21	0.32
Latency (minutes) to				
Sleep onset	13 [0–335]	14 [0–254]	0.49	0.51
REM sleep	120 [1–414]	128.5 [5–443]	0.34	0.55
Sleep duration (time in minutes)				
N 3 sleep	53 [0–144]	46 [0–166]	0.50	0.32
REM sleep	37 [0–153]	34 [0–140]	0.14	0.26
Sleep fragmentation (index per hour)				
Periodic legs movements during sleep	3.6 [0.0–123.4]	2.2 [0.0–200.3]	0.85	0.83(2)
Periodic legs movements with awakenings	0.5 [0.0–24.7]	0.3 [0.0–27.8]	0.72	0.44(2)
Arousals	15.6 [0.0–50.1]	16.9 [1.7–70.5]	0.18	0.38(2)
Apnea-hypopnea	7.0 [0.0–70.9]	7.6 [0.0–55.9]	0.83	0.82(2)
Mean oxygen saturation	95.0 [89.0–98.0]	95.0 [88.0–98.0]	0.11	0.27(2)
% of time with oxygen saturation <90%	0.08 [0.0–71.9]	0.04 [0.0–80.4]	0.39	0.26(2)
Multiple sleep latency tests				
Mean sleep latency	13.7 [2.8–20.0]	17.4 [4.4–20.0]	**0.007**	**0.03**
Mean sleep latency <8 min (%)	19.35	8.33	0.07	0.18

(1) after adjustment for body mass index;

(2) after adjustment for body mass index, stage 3 and REM sleep durations; continuous variables are expressed by median [Min–Max].

### Comparisons between PD patients with and without objective EDS

Only 18 patients (13.4%) were identified with objective daytime sleepiness (i.e. MSLT latency <8 minutes). Patients with objective sleepiness had a higher BMI and were more frequently overweight or obese than patients without sleepiness (83.3 vs 24.7%; p = 0.04) (Table 3). After adjustment for BMI, no significant between-group differences were found for demographic and clinical characteristics.

**Table 3:** Demographic and clinical characteristics of patients with Parkinson’s disease with objective daytime sleepiness (Multiple sleep latency tests-MSLT below 8 minutes) compared to patients with Parkinson’s disease without objective daytime sleepiness (MSLT above 8 minutes).

	MSLT latency			
Variables	<8 minN = 18	≥8 minN = 116	p	p(1)
Age (in years)	67.6±9.1	65.7±8.4	0.36	
Sex (% male)	83.33	69.83	0.25	
BMI (kg/m^2^)	27.6±3.5	24.7±4.0	**0.008**	
Motor disability				
UPDRS-III/108	27.6±17.3	21.0±11.9	0.12	0.13
Hoehn and Yahr	2.3±1.0	2.2±0.8	0.55	0.52
Age at onset (in years)	61.2±9.1	57.3±9.5	0.11	0.11
Disease duration (in years)	5.5 [1]–[15]	6 [1]–[23]	0.20	0.41
Restless legs syndrome (%)	16.67	33.62	0.16	0.19
Clinical RBD (%)	33.33	49.55	0.21	0.11
Insomnia severity	13.50±3.8	13.0±5.4	0.69	0.70
BDI score ≥19 (%)	25.00	28.04	0.82	0.99
Depression in the past (%)	33.33	25.64	0.62	0.58
MMSE score ≥26 (%)	93.33	87.61	0.52	0.58
Pain complaint (%)	57.14	77.03	0.26	0.23
Levodopa equivalent dose (mg/day)	850 [300–2158]	730 [67–2050]	0.07	0.08
intake before 12 AM	300 [100–750]	250 [0–725]	0.13	0.18
intake between 12 AM and 7 PM	300 [100–983]	250 [0–858]	0.11	0.09
intake after 7 PM	200 [0–533]	200 [0–550]	0.53	0.55
Dopamine agonists intake (%)	82.35	79.65	0.80	0.82
Antidepressants intake (%)	27.78	21.55	0.56	0.58
Benzodiazepines intake (%)	11.76	27.59	0.18	0.33

(1) after adjustment for body mass index; Continuous variables are expressed by mean ± standard deviation (SD) or median [Min–Max] according to the normality of the distribution.

Comparisons of nighttime sleep between PD patients with and without objective daytime sleepiness revealed a higher AHI in the former group even after further adjustments for BMI, N3 and REM sleep duration. No between-group differences were found for other sleep measures (Table 4). A severe obstructive SAS was more frequent in patients with objective sleepiness compared to those without (35.3 vs 15.7%; p = 0.05). The percentage of patients with severe SAS increased with BMI, being 9.7% in patients with normal BMI, 23.9% in overweight patients and 42.9% in obese patients.

**Table 4:** Night-time sleep and Multiple Sleep Latency Tests of patients with Parkinson’s Disease with objective sleepiness (Multiple sleep latency tests-MSLT below 8 minutes) compared to patients without (MSLT above 8 minutes).

	MSLT latency			
Variables	<8 min N = 18	≥8 min N = 116	p	p(1)
Night-time sleep				
Total sleep time (minutes)	349.5 [162–456]	310 [69–455]	0.38	0.61
Sleep efficiency	70 [19–95]	67 [16–91]	0.67	0.96
Latency (minutes) to				
Sleep onset	8 [0–173]	14 [0–335]	0.57	0.64
REM sleep	109 [8–414]	134 [1–443]	0.38	0.99
Sleep duration (time in minutes)				
Stage 3	56 [0–141]	50 [0–165.5]	0.80	0.54
REM sleep	33 [0–100]	35 [0–153]	0.91	0.39
Sleep fragmentation (index per hour)				
Periodic legs movements during sleep	1.1 [0.0–49.5]	3.6 [0.0–200.3]	0.32	0.42(2)
Periodic legs movements with awakenings	0.0 [0.0–2.8]	0.3 [0.0–27.8]	0.28	0.33(2)
Arousals	15.6 [5.9–25.1]	16.8 [0.0–70.5]	0.38	0.34(2)
Apnea-hypopnea	13.1 [0.9–70.9]	6.5 [0.0–55.9]	**0.01**	**0.04** (2)
Mean oxygen saturation	95.0 [91.6–97.0]	95.0 [88.0–98.0]	0.91	0.24(2)
% of time with oxygen saturation <90%	0.2 [0.0–2.5]	0.04 [0.0–80.4]	0.39	0.21(2)

(1) after adjustment for body mass index;

(2) after adjustment for body mass index, stage 3 and REM sleep durations; Continuous variables are expressed by mean ± standard deviation (SD) or median [Min–Max] according to the normality of the distribution.

In a sensitivity analysis, due to the limited number of patients included with a MSLT latency below 8 minutes which limits the power for the analysis, we chose to stratify patients according to the median MLST value of 10.2 minutes. Except for a higher BMI (p = 0.04), a tendency for higher levodopa equivalent dose (p = 0.06) and for higher AHI (12.6 [0.94–70.88] vs 5.9 [0.0–55.89], p = 0.07) in patients with MSLT<10.2 min, no other between-group differences were noted for demographic, clinical and polysomnographic parameters.

## Discussion

This is the largest study assessing both subjective and objective EDS in patients with PD unselected for sleep complaint. Self-reported EDS is frequent in PD reaching 46%, but objective sleepiness, defined by the gold standard MSLT, is less common (13.4%) with a weak correlation between ESS and MSLT. A large number of potential determinants and confounding factors for EDS were systematically assessed. Only a high BMI was associated with both subjective and objective sleepiness, pain with ESS and larger AHI with objective EDS.

The complaint of pain was found in 76% of patients with PD, a symptom associated with ESS with an odds ratio up to 4-fold. Pain is one of the most frequent non-motor symptoms reported in PD either during the day or at night together with nocturnal motor problems such as akinesia and dystonia. In addition to motor discomfort, pain in PD may also relate to an alteration in the regions involved in nociceptive pathways, depressive symptoms and sleep problems [22]. Our current knowledge about the relationship between pain and sleep is still sparse but sleep disturbances are frequently reported in patients with pain, and sleep-deprived conditions reduce the pain threshold and can even create pain de novo [23]. However we found no significant differences between patients with and without pain for PD severity, treatment, PSG parameters and depressive symptoms. In addition, no significant interaction was found between pain and BMI for the presence of EDS in PD patients.

Objective EDS was associated with a higher AHI even after adjustment for BMI, slow wave sleep and REM sleep duration. The latter adjustments were decided based on differences in respiratory event frequencies according to sleep duration and sleep stages. The frequency of SAS in PD and particularly in sleepy PD patients remains an issue of debate [3], [8], [11]. There are several possible explanations for this association, including age and BMI of the population, a potential loss of neurons involved in respiratory control and sleep physiology, and finally upper airway muscle dysfunction [24]. However, recent studies suggest that neither dopaminergic nor serotoninergic neuron degeneration are associated with the presence of OSAS in PD [25]. One recent study showed that treating OSAS with continous positive airway pressure reduces objective sleepiness in patients with PD [26]. Besides the general tendency for lower BMI in PD than in age-matched controls, in agreement with previous studies [10], [12], [14] we report associations between OSAS, BMI and MSLT in PD; but with no correlation found between ESS and AHI. However, we report associations between BMI and both ESS and MSLT, which are independent of OSAS. Our findings confirm the results obtained in both general population and clinical samples showing a close relationship between daytime sleepiness and obesity in subjects without sleep apnea [27]. The mechanisms underlying this association may relate to metabolic and proinflammatory disturbances, pathways of interest since both oxidative stress and inflammation are also potential players in the pathophysiology of PD [28].

Patients with PD taking psychotropic drugs may report EDS and as a result cause driving accidents, with a dose-response relationship regarding dopaminergic-induced sleepiness [1], . However, we report no significant association between PD severity, medication (i.e. dopamine agonists, levodopa, antidepressants and benzodiazepines) and either subjective or objective EDS, but only a tendency for a higher levodopa equivalent daily dose in patients with low MSLT latencies. Sleep and mood disorders were frequently found in PD such as nighttime sleep disruption, RLS, RBD, insomnia and depressive symptoms; however none were associated with EDS assessed clinically or through MSLT. In contrast to previous small or highly selected population studies, [3], [13] only rare patients had one SOREMP and none had more than one SOREMP, with no between-group differences for the presence of clinically or MSLT-defined EDS.

Altogether, our findings support the idea that EDS in PD is caused by multiple factors, some being disease-related while others are individually-driven. A poor correlation was found between ESS and MSLT indicating that both measures explored different components of sleepiness. ESS remains of real interest in clinical practice to screen the population at risk for daytime sleepiness even if patients with PD often misperceive their sleepiness which overlaps with the fatigue symptom [8], [12], [14]. A more structured clinical interview such as proposed in the DSM-5 criteria for hypersomnia disorder may be helpful in this context in assessing the presence, frequency, severity and phenotype of clinical excessive sleepiness in PD [29]. MSLT is the gold standard to evaluate daytime sleepiness in central hypersomnia; however most of studies comparing MSLT results in patients with PD to controls have failed to observe differences in mean sleep latencies [8], [11]. Even self-reported EDS dichotomized according to the widely taken but non validated cut-off score of 10 in PD ESS yielded in our study a sensitivity for MSLT below 8 min of 19.4% and a specificity of 91.7%, with corresponding positive and negative predictive values of 66.7 and 56.9%, respectively. Using a more severe ESS cut-off at 16 did not improve the diagnosis of objective EDS with a sensitivity of 23.5%, a specificity of 88%, with corresponding positive and negative predictive values of 22.2 and 88.8%, respectively. As MSLT seems unsatisfactory in the evaluation of EDS in PD, the use of alternative reliable measures such as the maintenance of wakefulness test (assessing the patient’s ability to maintain wakefulness rather than the drive to fall asleep) or the 24-h continuous polysomnography recording remains to be confirmed [12], [30]. All in all, the best method to explore objective EDS in PD remains unclear.

The study has several limitations. Firstly, as objective EDS assessed by MSLT is relatively rare in unselected PD patients, the limited number of patients included with objective EDS (n = 18) reduces the power for the analysis especially when reporting negative results. To show a significant association between patients with ESS<10 and patients with ESS≥10 and a mean sleep latency categorized into <8 min vs ≥8 min, 306 subjects would be necessary with a power of 0.80 and a type I error α = 0.05. Secondly, only one night of polysomnography-MSLT recording was performed in the sleep lab with potential for a first night effect. Thirdly, we adjusted for a large number of potential confounders for the presence of EDS; however data on driving accidents, hallucinations, physical and social activity, light exposure and comorbidities associated with overweight/obesity (i.e. diabetes, metabolic syndrome) were not available. We did not measure the sleep attacks (i.e. sleepiness without warning signs) being another rare phenotype difficult to assess and quantify in the sleep lab. The cross-sectional nature of the present study precluded any conclusion regarding the temporal sequencing between self-reported sleepiness, objective sleepiness, BMI, pain and OSAS. Finally we did not measure the key players that regulate sleep/wake and circadian systems in PD. However except for a recent study showing an association between low amplitude of the melatonin rhythm and EDS assessed through ESS in PD [31], the biological mechanism responsible for EDS in PD remains mostly unknown.

In conclusion, we found a high frequency of self-reported EDS in PD, a finding which was however not confirmed by a neurophysiological evaluation. Current treatment options for EDS are very limited in PD; it thus remained to be determined whether decreasing pain and BMI in association with the treatment of sleep apnea syndrome could decrease significantly daytime sleepiness in PD.
